# Optical Coherence Tomography Reveals Longitudinal Changes in Retinal Damage Under Different Treatments for Neuromyelitis Optica Spectrum Disorder

**DOI:** 10.3389/fneur.2021.669567

**Published:** 2021-07-19

**Authors:** Pei Zeng, Chen Du, Rui Zhang, Dongmei Jia, Feng Jiang, Moli Fan, Chao Zhang

**Affiliations:** ^1^Department of Neurology, Tianjin Neurological Institute, Tianjin Medical University General Hospital, Tianjin, China; ^2^Department of Ophthalmology, Tianjin Medical University General Hospital, Tianjin, China

**Keywords:** neuromyelitis optica spectrum disorder, retinal ganglion cells, azathioprine, tocilizumab, rituximab

## Abstract

**Background:** Progressive retinal neuroaxonal damage after acute optic neuritis may occur in neuromyelitis optica spectrum disorder (NMOSD). However, it is unclear if treatments used to prevent attacks influence neurodegeneration.

**Objectives:** We aimed to investigate retinal damage in patients treated with disease-modifying drugs in a longitudinal study.

**Methods:** We retrospectively included 50 patients with aquaporin 4-antibody-seropositive NMOSD. Peripapillary retinal nerve fiber layer (pRNFL) thickness, macular ganglion cell complex (mGCC) thickness, total macular volume (TMV), and optic disc measures were acquired by spectral domain optical coherence tomography in patients treated with tocilizumab, rituximab, and azathioprine.

**Results:** Longitudinally, in eyes with a history of ON (NMOSD^ON+^), we observed annual thinning of mGCC [tocilizumab: −1.77 (−3.44, −0.09) μm, *p* = 0.041; rituximab: −2.03 (−3.58, −0.48) μm, *p* = 0.017; azathioprine: −1.79 (−2.22, −1.37) μm, *p* < 0.001], and pRNFL [tocilizumab: −2.07 (−0.75, −3.39) μm, *p* = 0.005; rituximab: −2.18 (−0.36, −4.00) μm, *p* = 0.023; azathioprine: −2.37 (−0.98, −3.75) μm, *p* = 0.003], reduced TMV [tocilizumab: −0.12 (−0.22, −0.01) mm^3^, *p* = 0.028; rituximab: −0.15 (−0.21, −0.08) mm^3^, *p* = 0.001; azathioprine: −0.12 (−0.20, −0.04) mm^3^, *p* = 0.006], and increased cup area [tocilizumab: 0.08 (−0.01, 0.16) mm^2^, *p* = 0.010; rituximab: 0.07 (0.01, 0.12) mm^2^, *p* = 0.019; azathioprine: 0.14 (0.02, 0.26) mm^2^, *p* = 0.023]. However, we detected no significant differences in annual changes in mGCC, pRNFL, TMV, and cup area between patients with tocilizumab, rituximab, and azathioprine in NMOSD^ON+^ eyes. NMOSD^ON−^ eyes did not display mGCC or pRNFL thinning in patients treated with tocilizumab and rituximab. Intriguingly, we observed significant thinning of mGCC in patients treated with azathioprine compared with tocilizumab [−0.84 (−1.50, −0.18) μm vs. −0.19 (−0.87, 0.48) μm, *p* = 0.012] and rituximab [−0.84 (−1.50, −0.18) μm vs. −0.07 (−1.25, −2.51) μm, *p* = 0.015] in NMOSD^ON−^ eyes.

**Conclusions:** This study demonstrated that retinal ganglion cell loss is independent of ON attacks in NMOSD. Tocilizumab and rituximab may delay mGCC thinning in NMOSD^ON−^ eyes compared with azathioprine.

## Introduction

Neuromyelitis optica spectrum disorder (NMOSD) is a debilitating humoral-mediated autoimmune disease, which is typically characterized by attacks of acute optic neuritis (ON) and longitudinally extensive transverse myelitis (1). Inflammation of the optic nerve and impaired axonal transport are associated with severe visual dysfunction during acute ON. Retinal ganglion cell and axonal loss after acute ON in NMOSD have been detected extensively by the potential use of quantitative optical coherence tomography (OCT). Neuronal thinning was observed in the ganglion cell layer in ON-affected eyes even within 3 and 6 months. Only minimal retinal nerve fiber layer thickness (RNFL) loss was observed after this period (2, 3).

Recent studies indicate that retinal ganglion cell loss may persist and be independent of ON attacks in patients with NMOSD with seropositive anti-aquaporin 4 antibody (AQP4-IgG) over a 2-year follow-up (4–7).

Progressive ganglion cell layer thinning may be partly due to retrograde neuroaxonal degeneration from lesions or primary retinopathy (8, 9). In addition, different from ON in patients with multiple sclerosis (MS), patients with NMOSD exhibit greater reductions in RNFL thickness and average macular volume (10–12). Several molecular pathways have been identified as potential therapeutic targets to induce remyelination after ON in patients with MS (13). However, to date, few therapies for NMOSD improve remyelination directly. Empirical treatments including azathioprine and B cell-depleting treatments have been extensively used to prevent ON attacks (14, 15). Recently, IL-6 receptor monoclonal antibody satralizumab and tocilizumab have demonstrated a reduced risk of attacks in NMOSD (16, 17). However, it remains unclear whether these drugs can prevent or delay neurodegeneration in eyes with and without a history of ON in patients with NMOSD.

In this study, we aimed to investigate longitudinal changes of OCT measures and compare them in patients with NMOSD under different treatments, including tocilizumab, rituximab, and azathioprine.

## Materials and Methods

### NMOSD Patients and Healthy Controls

One hundred and twenty-one patients with NMOSD from the research database of the Department of Neurology, Tianjin Medical University General Hospital, were screened. Inclusion criteria included the following: (1) a confirmed diagnosis of AQP4-IgG seropositive NMOSD according to the 2015 international consensus criteria (18); (2) complete longitudinal clinical and OCT imaging data with a minimum follow-up of 1 year; and (3) age between 18 and 75 years at baseline. Patients with any of the following conditions were excluded from the study: (1) < 6 months after ON onset; (2) experienced a new ON attack during follow-ups; (3) spherical equivalent > 2 diopters; (4) intraocular pressure > 21 mmHg; (5) history of other ocular diseases other than cataract, such as glaucoma, uveitis, or retinal diseases; (6) history of ocular surgery, laser treatment, or ocular trauma; (7) systemic diseases, such as diabetes mellitus, systemic hypertension, Parkinson's disease, and Alzheimer's disease; or (8) severe ONs that may cause difficulty in fixation for OCT examination.

For comparison, we collected longitudinal data for 20 matched eyes from 10 healthy controls (HCs) with equal median duration of follow-up. None of the HCs had a history of disease or inflammation and none received any treatments before the study and during follow-ups.

### Standard Protocol Approvals, Registrations, and Patient Consents

The study was approved by the Institute Ethical Board of Tianjin Medical University General Hospital and was conducted in accordance with the Declaration of Helsinki. Written informed consent was obtained from each participant before the study.

### Visual Acuity Measurements

To assess vision precisely, low-contrast letter acuity was measured using retro-illuminated 2.5% Sloan letter chart (Precision Vision, La Salle, IL, USA) with best refractive monocular correction at 2.52 m. The maximal number of letters visible on this chart was 70. Best-corrected high-contrast logMAR visual acuity was measured using retro-illuminated Early Treatment Diabetic Retinopathy Study chart (Precision Vision, La Salle, IL, USA) at 2.52 m. When no letters could be correctly identified, a score of 1.7 was assigned (19).

### Spectral-Domain Optical Coherence Tomography

High-resolution spectral-domain optical coherence tomography (SD-OCT) images were acquired at baseline and the last follow-up using identical protocols (RTVUE100-2, Optovue Inc., Fremont, CA, USA). We used the Auto buttons for the first measurement for each eye to fix scanning. After the first scan was optimized and captured, the system stored the settings for each eye and the same settings were used for all subsequent visits. The OCT Image acquisition rate was 26,000 A-scans/s with 1024 A-scan for each frame. The in-tissue optical resolution was 5 μm in depth and 15 μm in beam spot size. We used ONH and GCC (ganglion cell complex) scan patterns for measurements. The ONH mode was used to get several pieces of important disc morphology information, including disc and cup areas, C/D ratio, and RNFL 3.45 thickness map. The ONH protocol used 12 radial line scans (each composed of 12 × 455 A-scans) with 3.4-mm length and 13 concentric rings (each composed of 3 × 965 A-scans). Software available on this machine automatically detects the center of the optic disc and provides the mean RNFL around a circumpapillary circle with a 3.45-mm radius from the center of the optic disc. The GCC mode was used to get mGCC thickness. The GCC protocol adopted 1 horizontal line (1 × 933 A-scans) with 7 mm scan length, followed by 15 vertical lines (each composed of 15 × 933 A-scans) with 7 mm scan length and 0.5 mm interval, centered 1 mm temporal to fovea. An auto average model was set to calculating the average of repeated B-scans to help reduce the possibility of test–retest variability.

In the workstation of the OCT instrument, the macular GCC (mGCC) indicates the combination of macular RNFL, ganglion cell layer, and inner plexiform layer. We also measured the focal loss volume (FLV) and global loss volume (GLV). FLV is a parameter that provides a quantitative determination of the amount of significant GCC loss. It is the total sum of significant GCC loss (in volume) divided by the map area. GLV is the sum of the pixels where the Fractional Deviation map value is <0 and then divided by the total area to give a percent loss of GCC thickness.

The peripapillary RNFL (pRNFL) was measured with activated eye tracker using the innermost 3.45 mm ring of a star-and-ring scan around the center of the disc. Total macular volume (TMV) was calculated as a 6-mm-diameter cylinder around the fovea from a macular volume scan. The fovea thickness (FT) was defined as the mean thickness of the 1-mm-diameter cylinder around the fovea from the same scan. Optic disc measurements included optic disc area, cup area, rim area, cup volume, rim volume, and nerve head volume. We adhered to the APOSTEL criteria when reporting OCT results (20). An example of the scanning procedure in one eye of a patient with NMOSD is shown in Supplementary Figures 1–3.

One experienced rater carefully checked all scans for sufficient quality and segmentation errors and corrected them when necessary. The OSCAR-IB quality control criteria were used to report OCT data quality and exclusions (21). Seven eyes were excluded because of insufficient data quality and severe ONs that rendered patients unable to cooperate with the scanning.

### Intervention of the Patients With NMOSD

Patients with tocilizumab received infusions at the dose of 8 mg/kg/month and patients with azathioprine received oral treatment at the dose of 2–3 mg/kg per day. Patients with rituximab received periodical treatment according to the proportion of B cells in peripheral blood mononuclear cells (PBMCs), as previously published (22). Briefly, the percentage of circulating CD19+ B cells in the PBMCs was determined. The doses of rituximab were administered to the patients according to the CD19+ B cell counts: (1) 0.5–1.5% of PBMCs, 100 mg of rituximab was administered intravenously once; (2) 1.5–5.0% of PBMCs, 100 mg of rituximab was administered intravenously for 2 consecutive days; (3) 5.0–15.0% of PBMCs, 100 mg of rituximab was administered intravenously for 3 consecutive days; and (4) >15.0% of PBMCs, 100 mg of rituximab was administered intravenously for 4 consecutive days. The percentage of CD19+ B cells was monitored at 12-week intervals. The indicated dose reinfusion regimen was based on CD19+ B-cell repopulation, when the circulating CD19+ B-cell proportion >0.5% of PBMCs.

### Statistical Analysis

Group differences between NMOSD patients and HCs were tested by χ^2^ test for sex and Wilcoxon rank-sum test for age. The primary outcome was the change in mGCC over the follow-ups; secondary outcomes were changes in pRNFL, FT, TMV, and visual acuity. Cross-sectional differences of OCT values and visual acuity among all groups were analyzed pairwise. Annual loss was estimated for each eye as change from baseline at last visit divided by the follow-up time (years). Taking inter-eye correlation into account, we performed a generalized estimating equation (GEE) analysis with changes of retinal index as the dependent variable and treatments as predictors. This model tested the interaction between predictors. Comparisons of mGCC, pRNFL, TMV, and FT between different treatment groups were performed by GEE. The Wald *p*-value, regression coefficient (β), and 95% confidence interval (CI) are reported. All tests and graphical representations were performed with SPSS v23.0 (IBM SPSS, Armonk, NY, USA) in accordance with APOSTEL. Statistical significance was established at *p* < 0.05.

## Results

### Cohort Description and Follow-Up

Among the 121 patients with NMOSD screened, data for 50 patients with NMOSD with a mean follow-up time of 1.18 (1.01) years fulfilled the inclusion criteria: 20 patients received tocilizumab treatment, 18 patients received azathioprine treatment, and 12 patients received rituximab treatment. Of all patients with NMOSD, 45 experienced unilateral ON, and 5 patients experienced bilateral ON. Thus, 55 eyes experienced a history of ON (NMOSD^ON+^) and 45 eyes had no history of ON (NMOSD^ON−^). Visual acuity and Expanded Disability Status Scale assessment at baseline are available in Table 1. Sex, age, follow-up time, disease duration, ARR, EDSS, eyes with a history of ON, ON episodes per ON eye, time since last ON, LogMAR visual acuity, and 2.5% low-contrast letter acuity were comparable between the patients treated with tocilizumab, rituximab, and azathioprine (*p* > 0.05).

**Table 1:** Demographic characteristics of NMOSD patients and HCs.

	**Tocilizumab**	**Rituximab**	**Azathioprine**	**HCs**
Subject (*N*)	20	12	18	10
Sex (female in %)	20 (100)	11 (91.67)	18 (100)	9 (90)
Age at baseline (years, mean ± SD)	41.35 ± 17.52	53.25 ± 13.09	41.28 ± 14.61	44.43 ± 12.90
Follow-up time [days, median (IQR)]	403.5 (307.5–617.3)	390 (263.3–552.5)	438.50 (173.5–619.5)	412.0 (323.5–667.5)
Disease duration at baseline (years, mean ± SD)	5.85 ± 2.81	5.78 ± 2.53	5.61 ± 2.58	/
Time on current treatment [months, median (IQR)]	8.68 (7.3–10.5)	8.45 (6.3–11.5)	8.89 (6.3–9.33)	/
ARR before baseline (mean ± SD)	1.18 ± 0.97	1.23 ± 1.11	1.65 ± 1.37	/
EDSS at baseline [median (IQR)]	3.5 (2.3–6.5)	3.8 (2.5–6.5)	3.5 (3.3–7.0)	/
Eyes with a history of ON (*N*, %)	22 (55.0%)	12 (50.0%)	21 (58.3%)	/
ON episodes per ON eye (mean ± SD)	1.0 ± 0.3	1.0 ± 0.4	1.1 ± 0.3	/
Tine since last ON [months, median (IQR)]	8.2 (6.3–9.7)	8.7 (7.3–9.8)	8.3 (6.5–9.3)	/
LogMAR visual acuity of NMOSD^ON+^ eyes (median, IQR)	0.72 (0.71–0.76)	0.69 (0.68–0.71)	0.73 (0.70–0.75)	0.10 (0–0.15)
2.5% low-contrast letter acuity of NMOSD^ON+^ eyes (median, IQR)	6.8 (6.3–6.9)	6.7 (6.5–7.0)	7.1 (6.9–7.3)	23.6 (22.8–24.3)
LogMAR visual acuity of NMOSD^ON−^ eyes (median, IQR)	0.17 (0.14–0.19)	0.16 (0.16–0.19)	0.18 (0.17–0.20)	/
2.5% low-contrast letter acuity of NMOSD^ON−^ eyes (median, IQR)	23.5 (22.3–25.0)	23.5 (22.1–25.3)	24.3 (23.3–25.7)	/

*ARR, annualized relapsing rate; EDSS, the Kurtzke Expanded Disability Status Scale; HCs, healthy controls; NMOSD, neuromyelitis optica spectrum disorder; ON, optic neuritis; SD, standard deviation. The patients between tocilizumab, rituximab, and azathioprine groups are comparable in all the parameters (p > 0.05)*.

### Baseline Characteristics of Visual Acuity and OCT Measures

At baseline, the LogMAR visual acuity of NMOSD^ON+^ eyes (0.76, IQR 0.73–0.78) was significantly lower than that of HCs eyes (0.10, IQR 0–0.15, *p* = 0.037). The 2.5% low-contrast letter acuity of NMOSD^ON+^ eyes (6.9, IQR 6.6–7.1) was also significantly lower than that of HCs eyes (23.8, IQR 22.8–24.6, *p* < 0.001). The LogMAR visual acuity of NMOSD^ON−^ eyes (0.18, IQR 0.16–0.19) did not differ significantly from that of HCs eyes (*p* = 0.438). The 2.5% low-contrast letter acuity of NMOSD^ON−^ eyes (23.5, IQR 22.5–24.5) was not lower than that of HCs eyes (*p* = 0.983) (Table 2).

**Table 2:** Baseline visual acuity and OCT measures NMOSD eyes and HCs eyes.

	**Eyes^**ON+**^**	**Eyes^**ON−**^**	**HCs**
**LogMAR visual acuity**	0.76 (0.73–0.78)	0.18 (0.16–0.19)	0.10 (0–0.15)
**2.5% low-contrast letter acuity**	6.9 (6.6–7.1)	23.5 (22.5–24.5)	23.8 (22.8–24.6)
**mGCC thickness (μm)**	66.91 (8.23)	91.92 (3.52)	97.53 (2.32)
Superior	79.11 (16.33)	91.15 (6.13)	94.35 (4.21)
Inferior	69.45 (9.97)	90.91 (5.72)	94.37 (3.67)
FLV (%)	6.83 (4.54)	1.16 (1.63)	0.45 (0.49)
GLV (%)	25.19 (9.66)	6.69 (4.62)	4.59 (1.85)
**Global pRNFL (μm)**	71.88 (12.94)	110.50 (6.94)	112.40 (8.10)
**Macular**			
TMV (mm^3^)	6.30 (0.30)	6.93 (0.27)	7.23 (0.37)
FT (μm)	227.57 (13.13)	240.47 (13.01)	252.73 (18.58)
**Optic disc**			
Disc area (mm^2^)	2.36 (0.51)	2.14 (0.33)	2.25 (0.38)
Cup area (mm^2^)	1.19 (0.67)	0.62 (0.31)	0.61 (0.37)
Cup volume (mm^3^)	0.24 (0.17)	0.10 (0.07)	0.10 (0.09)
Rim area (mm^2^)	1.18 (0.44)	1.52 (0.28)	1.70 (0.34)
Rim volume (mm^3^)	0.08 (0.07)	0.18 (0.07)	0.23 (0.12)
Nerve head volume (mm^3^)	0.19 (0.12)	0.35 (0.13)	0.44 (0.23)

*Visual acuity was shown using median (IQR), and other data were shown using mean (SD). FLV, focal loss volume; FT, fovea thickness; GLV, global loss volume; HCs, healthy controls; mGCC, macular ganglion cell complex; pRNFL, peripapillary retinal nerve fiber layer thickness; TMV, total macular volume*.

We detected significant differences in OCT measures between patients with NMOSD and HCs (Table 2). In patients with NMOSD at baseline, the mGCC thickness in both NMOSD^ON+^ eyes (66.91 ± 8.23 μm, *p* < 0.001) and NMOSD^ON−^ eyes (91.92 ± 3.52 μm, *p* < 0.001) was significantly lower than that of HCs eyes (97.53 ± 2.32 μm). The mGCC thickness in NMOSD^ON+^ eyes was also lower than that of NMOSD^ON−^ eyes (mean difference 25.00 ± 1.64 μm, *p* < 0.001). Similarly, both the superior hemisphere mGCC thickness (79.11 ± 16.33 μm) and inferior hemisphere mGCC thickness (69.45 ± 9.97μm) of NMOSD^ON+^ eyes were significantly lower than those of HCs eyes (94.35 ± 4.21 μm, *p* = 0.043; 94.37 ± 3.67, *p* < 0.001, respectively) and NMOSD^ON−^ eyes (91.15 ± 6.13 μm, *p* = 0.012; 90.91 ± 5.72 μm, *p* < 0.001, respectively). Also, the thickness in the superior hemisphere (91.15 ± 6.13 μm, *p* = 0.043) and inferior hemisphere (90.91 ± 5.72 μm, *p* = 0.036) of NMOSD^ON−^ eyes was significantly lower than that in HCs eyes (94.35 ± 4.21 μm). The FLV% in NMOSD^ON+^ eyes (6.83 ± 4.54) was significantly higher than that in NMOSD^ON−^ eyes (1.16 ± 1.63, *p* < 0.001) and in HCs eyes (0.45 ± 0.49, *p* < 0.001); however, FLV% did not differ between NMOSD^ON−^ eyes and HCs eyes. The GLV% in NMOSD^ON+^ eyes (25.19 ± 9.66) was significantly higher than that in NMOSD^ON−^ eyes (6.69 ± 4.62, *p* < 0.001) and HCs eyes (4.59 ± 1.85, *p* < 0.001), and there was no difference in the GLV% between NMOSD^ON−^ eyes and HCs eyes.

Besides, pRNFL thickness was significantly reduced in NMOSD^ON+^ eyes (71.88 ± 12.94 μm, *p* < 0.001) compared to HCs eyes (112.40 ± 8.10 μm), but there was no significant reduction in NMOSD^ON−^ eyes (110.50 ± 6.94 μm, *p* = 0.391).

In addition, TMV in NMOSD^ON+^ eyes (6.30 ± 0.30 mm^3^) was significantly reduced compared with that in NMOSD^ON−^ eyes (6.93 ± 0.27 mm^3^, *p* < 0.001) and HCs eyes (7.23 ± 0.37 mm^3^, *p* < 0.001). TMV in NMOSD^ON−^ eyes was also lower than that in HCs eyes (*p* = 0.005). Both NMOSD^ON+^ eyes (227.57 ± 13.13 μm, *p* < 0.001) and NMOSD^ON−^ eyes (240.47 ± 13.01 μm, *p* = 0.018) showed lower FT than HCs eyes (252.73 ± 18.58 μm).

Although, disc area in NMOSD^ON+^ eyes (2.36 ± 0.51 mm^2^) was significantly greater than that in NMOSD^ON−^ eyes (2.14 ± 0.33 mm^2^, *p* = 0.018), it was only slightly, but not significantly, increased in HCs eyes (2.25 ± 0.38 mm^2^, *p* = 0.484). NMOSD^ON+^ eyes displayed significantly increased cup area (1.19 ± 0.67 mm^2^) compared to NMOSD^ON−^ eyes (0.62 ± 0.31 mm^2^, *p* < 0.001) and HCs eyes (0.61 ± 0.37 mm^2^, *p* = 0.001). Correspondingly, cup volume in NMOSD^ON+^ eyes (0.24 ± 0.17 mm^3^) also increased compared with NMOSD^ON−^ eyes (0.10 ± 0.07 mm^3^, *p* < 0.001) and HCs eyes (0.10 ± 0.09 mm^3^, *p* = 0.006). However, NMOSD^ON+^ eyes demonstrated significantly decreased rim area (1.18 ± 0.44 mm^2^), rim volume (0.08 ± 0.07 mm^3^), and nerve head volume (0.19 ± 0.12 mm^3^) compared to NMOSD^ON−^ eyes (1.52 ± 0.28 mm^2^, *p* < 0.001; 0.18 ± 0.07 mm^3^, *p* < 0.001; 0.35 ± 0.13 mm^3^, *p* < 0.001) and HCs eyes (1.70 ± 0.34 mm^2^, *p* = 0.001; 0.23 ± 0.12 mm^3^, *p* = 0.001; 0.44 ± 0.23 mm^3^, *p* = 0.002). Although, NMOSD^ON−^ eyes demonstrated a reduction in rim area, rim volume, and nerve head volume, there were no significant differences between NMOSD^ON−^ eyes and HCs eyes.

**Figure 1:**
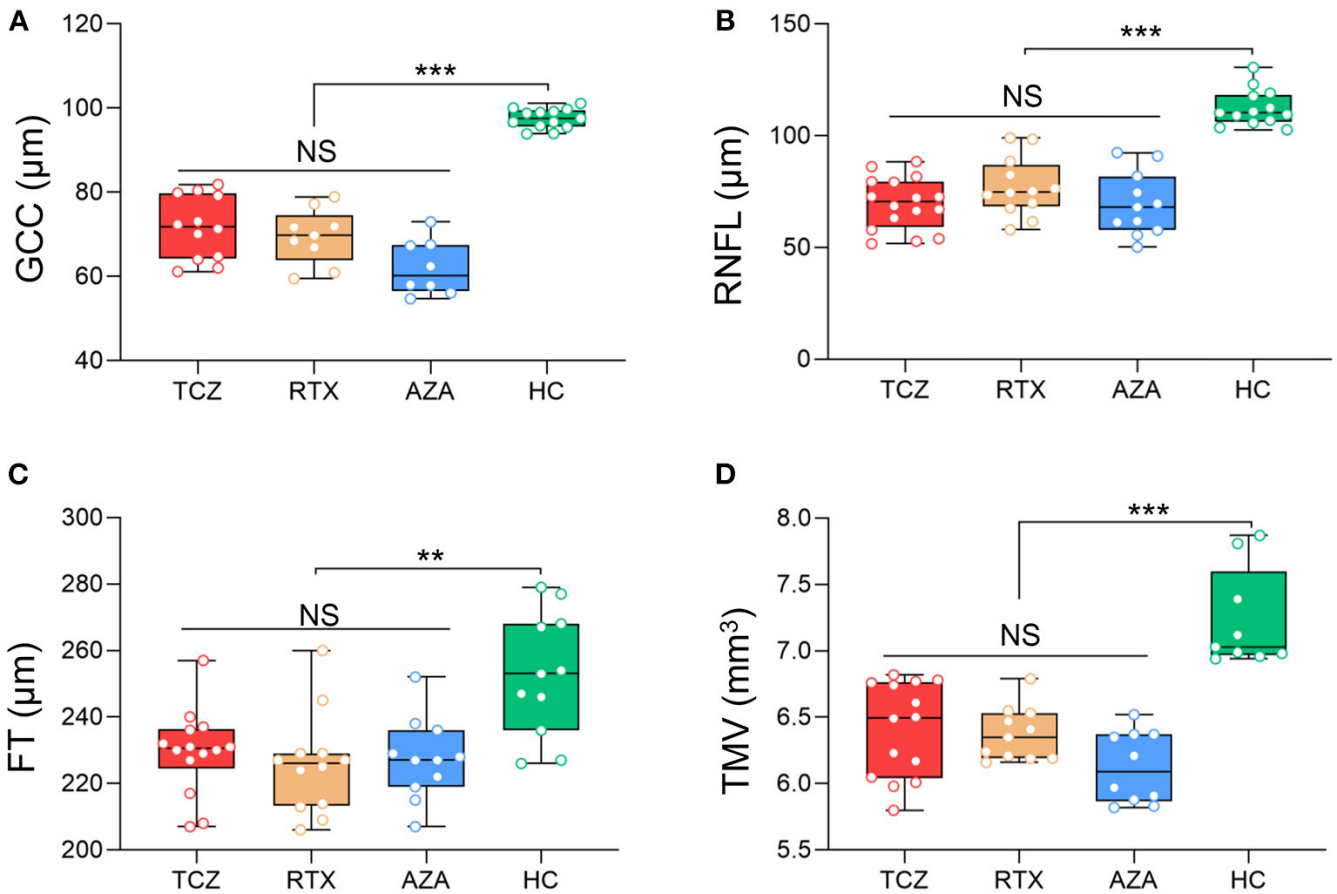
Baseline SD-OCT measures in NMOSDON+ eyes in patients with NMOSD. Mean thickness of the macular GCC **(A)**, RNFL **(B)**, FT **(C)**, and TMV **(D)** in eyes with a history of ON were measured by SD-OCT at baseline. GCC, ganglion cell complex; RNFL, retinal nerve fiber layer thickness; FT, fovea thickness; TMV, total macular volume; TCZ, tocilizumab, *n* = 19; RTX, rituximab, *n* = 12; AZA, azathioprine, *n* = 18; HC, healthy control, *n* = 20. **p < 0.01, ***p < 0.001. NS, not significant.

**Figure 2:**
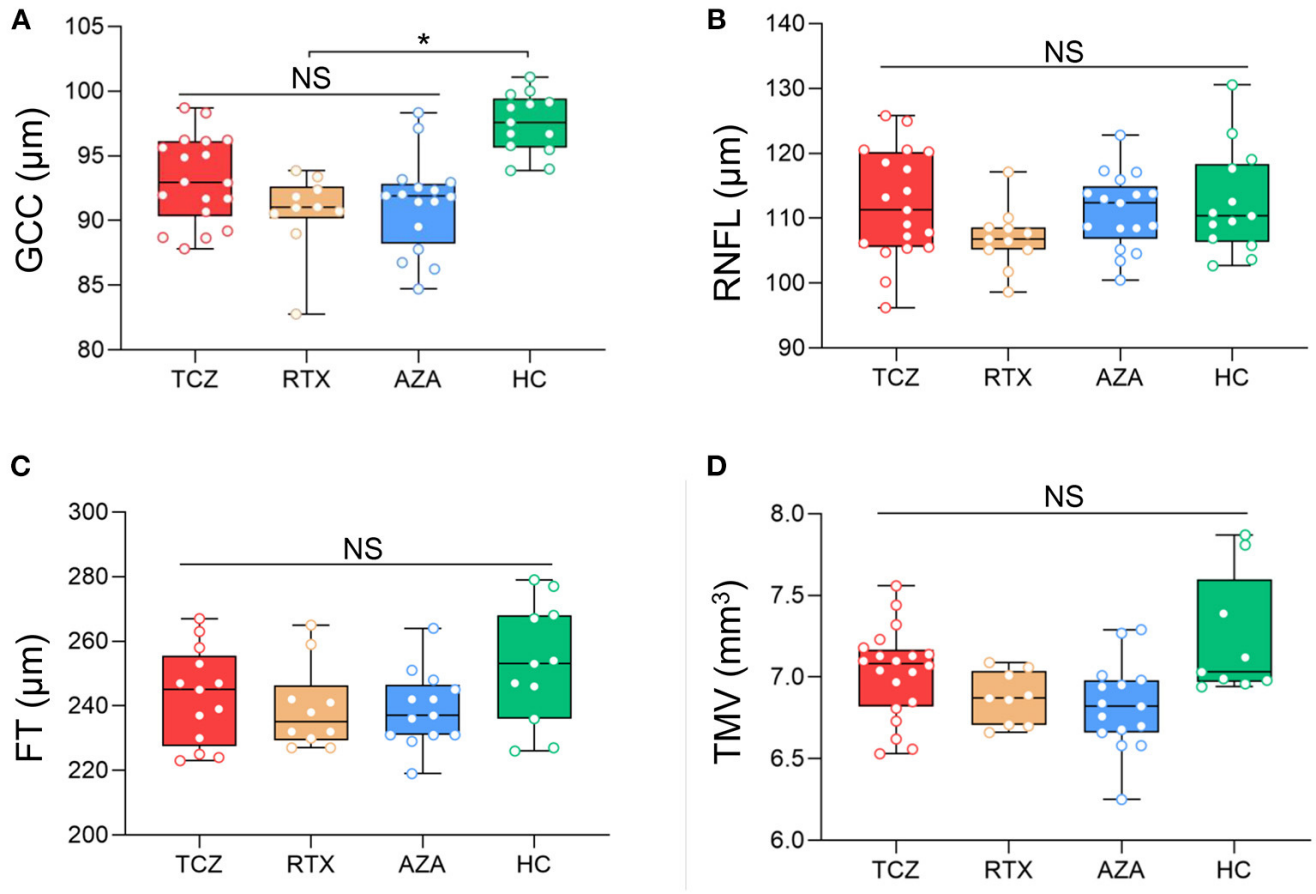
Baseline SD-OCT measures in NMOSDON− eyes in patients with NMOSD. Mean thickness of the macular GCC **(A)**, RNFL **(B)**, FT **(C)**, and TMV **(D)** in eyes without a history of ON were measured by SD-OCT at baseline. GCC, ganglion cell complex; RNFL, retinal nerve fiber layer thickness; FT, fovea thickness; TMV, total macular volume; TCZ, tocilizumab, *n* = 18; RTX, rituximab, *n* = 11, AZA, azathioprine, *n* = 15; HC, healthy control, *n* = 20. *p < 0.05. NS, not significant.

All OCT measures were collected at baseline for the three groups of medications (tocilizumab, rituximab, and azathioprine) and showed no significant differences, in either NMOSD^ON+^ or NMOSD^ON−^eyes (Figures 1, 2 and Supplementary Table 1).

### Visual Acuity Changes During Follow-Ups

We did not detect any changes in LogMAR visual acuity or low-contrast letter acuity either in either NMOSD^ON+^ eyes or in NMOSD^ON−^ eyes.

### Changes of OCT Measures in NMOSD^ON+^ Eyes During Follow-Ups

Longitudinally, we identified mGCC annual thinning in NMOSD^ON+^ eyes in the tocilizumab group (annual thinning −1.77 [−3.44, −0.09] μm, *p* = 0.041), the rituximab group (annual thinning −2.03 [−3.58, −0.48] μm, *p* = 0.017), and the azathioprine group (annual thinning −1.79 [−2.22, −1.37] μm, *p* < 0.001), compared with the baseline for each subgroup. This effect remained significant for each group relative to HCs. We also observed a thinning of pRNFL in NMOSD^ON+^ eyes compared with the baseline among the three groups, respectively: tocilizumab group (annual thinning −2.07 [−0.75, −3.39] μm, *p* = 0.005); rituximab group (annual thinning −2.18 [−0.36, −4.00] μm, *p* = 0.023); azathioprine group (annual thinning −2.37 [−0.98, −3.75] μm, *p* = 0.003). Significant thinning of pRNFL for each group against HCs was observed. Compared with the baseline in each group, all patients had significant annual TMV loss in NMOSD^ON+^ eyes: tocilizumab (annual loss −0.12 [−0.22, −0.01] μm, *p* = 0.028), azathioprine (annual loss −0.12 [−0.20, −0.04] μm, *p* = 0.006), and rituximab (annual loss −0.15 [−0.21, −0.08] μm, *p* = 0.001) (Table 3).

**Table 3:** Mean changes in the eyes with a history of ON in SD-OCT measures in patients with NMOSD and HCs.

	**Tocilizumab**		**Rituximab**		**Azathioprine**		**HCs**	
	**Eyes^**ON+**^**	***p***	**Eyes^**ON+**^**	***p***	**Eyes^**ON+**^**	***p***	**Eyes**	***p***
**mGCC thickness (μm)**	−1.77 (−3.44, −0.09)	**0.041**	−2.03 (−3.58, −0.48)	**0.017**	−1.79 (−2.22, −1.37)	** <0.001**	0.00 (−0.31, 0.32)	0.980
Superior	−0.37 (−7.32, 2.68)	0.706	0.33 (−4.59, 1.98)	0.569	−0.84 (−3.01, 3.01)	0.578	1.40 (−0.51, 2.01)	0.151
Inferior	−0.44 (−3.45, 1.45)	0.561	−0.87 (−2.95, 1.40)	0.301	−0.01 (−1.91, 1.28)	0.742	0.05 (−1.13,1.14)	0.970
FLV (%)	−0.66 (−2.26, 0.94)	0.392	−6.15 (−6.65, −4.34)	**0.001**	1.20 (−2.40, 4.80)	0.471	0.01 (−0.26, 0.27)	0.965
GLV (%)	1.15 (−1.90, 4.19)	0.435	0.91 (−0.78, 2.60)	0.261	0.06 (−1.38, 1.50)	0.928	−0.16 (−0.80, 0.48)	0.587
**Global pRNFL (μm)**	−2.07 (−0.75, −3.39)	**0.005**	−2.18 (−0.36, −4.00)	**0.023**	−2.37 (−0.98, −3.75)	**0.003**	−0.23 (−1.69, 2.15)	0.796
**Macular**								
TMV (mm^3^)	−0.12 (−0.22, −0.01)	**0.028**	−0.15 (−0.21, −0.08)	**0.001**	−0.12 (−0.20, −0.04)	**0.006**	0.00 (−0.06,0.04)	0.738
FT (μm)	−0.50 (−5.72, 4.72)	0.839	−0.50 (−7.93, 6.93)	0.885	−2.36 (−5.04, 0.31)	0.077	−0.27 (−1.07, 0.53)	0.465
**Optic disc**								
Disc area (mm^2^)	0.00 (−0.01, 0.01)	0.706	0.01 (−0.01, 0.03)	0.266	0.00 (−0.03, 0.01)	0.777	−0.01 (−0.02, 0.01)	0.391
Cup area (mm^2^)	0.08 (−0.01, 0.16)	**0.010**	0.07 (0.01, 0.12)	**0.019**	0.14 (0.02, 0.26)	**0.023**	0.03 (−0.01, 0.07)	0.129
Cup volume (mm^3^)	0.03 (0.00, 0.06)	**0.038**	0.05 (0.016, 0.08)	**0.006**	0.02 (−0.00, 0.05)	**0.020**	0.01 (−0.03, 0.05)	0.588
Rim area (mm^2^)	−0.10 (−0.21, 0.02)	0.103	−0.07 (−0.12, 0.02)	0.050	−0.14 (−0.27, −0.02)	**0.024**	−0.05 (−0.11, 0.01)	0.087
Rim volume (mm^3^)	−0.01 (−0.02, 0.000)	**0.031**	−0.01 (−0.02, 0.000)	0.028	−0.01 (−0.02, 0.00)	**0.041**	−0.01 (−0.02, 0.00)	0.055
Nerve head volume (mm^3^)	−0.01 (−0.06, 0.02)	0.247	−0.02 (−0.06, 0.00)	0.050	−0.02 (−0.06, 0.00)	0.050	−0.01 (−0.06, 0.02)	0.068

*The differences were shown using B (lower CI, upper CI). FLV, focal loss volume; FT, fovea thickness; GLV, global loss volume; HCs, healthy controls; mGCC, macular ganglion cell complex; pRNFL, peripapillary retinal nerve fiber layer thickness; TMV, total macular volume. p-values that were < 0.05 are in bold*.

**Figure 3:**
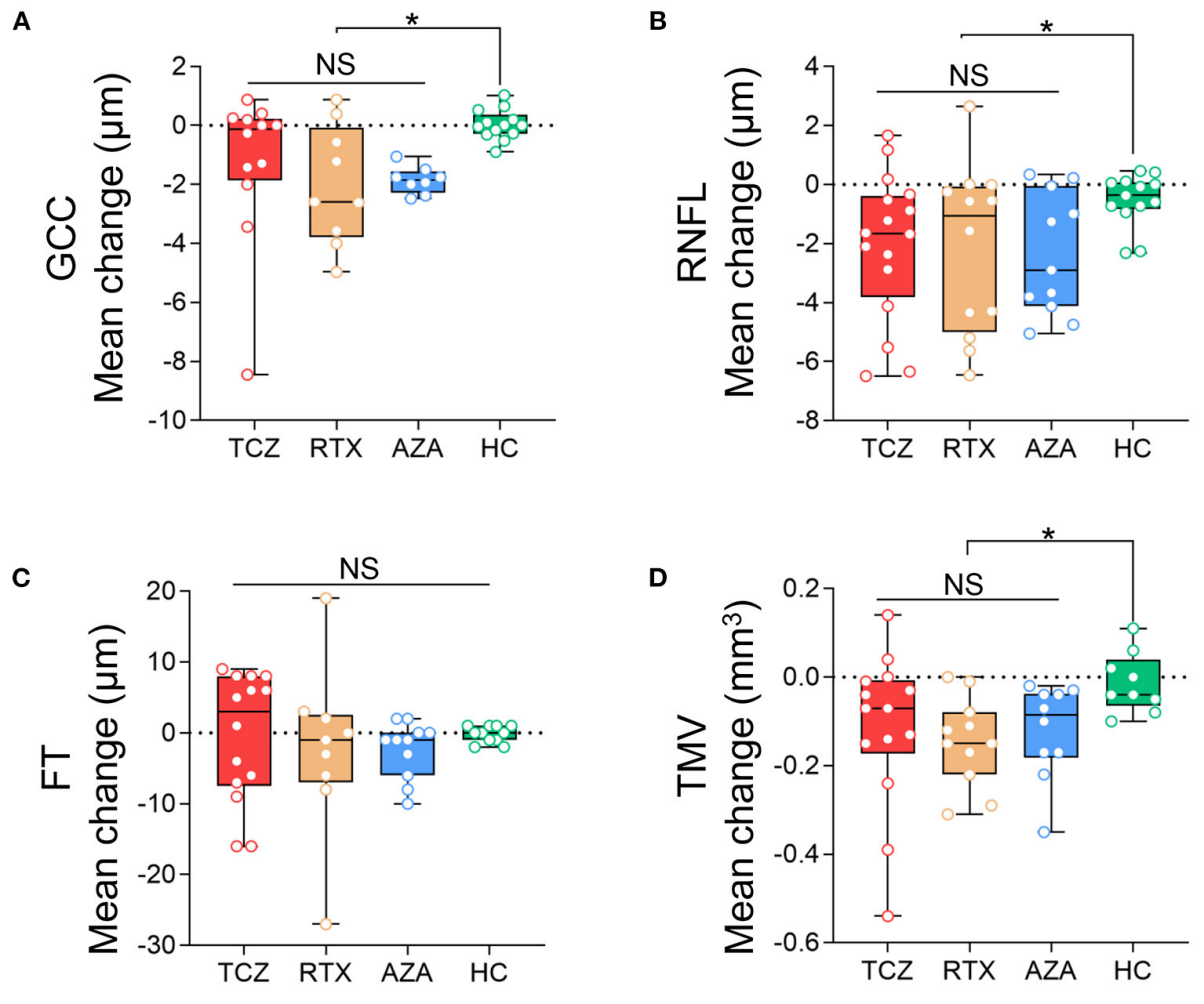
Comparison of the annual changes of SD-OCT measurements in NMOSDON+ eyes in patients with NMOSD. Annual loss of the macular GCC **(A)**, RNFL **(B)**, FT **(C)**, and TMV **(D)** in eyes with a history of ON were compared between each two groups. GCC, ganglion cell complex; RNFL, retinal nerve fiber layer thickness; FT, fovea thickness; TMV, total macular volume; TCZ, tocilizumab; RTX, rituximab; AZA, azathioprine; HC, healthy control. *p < 0.05. NS, not significant.

We observed no significant differences in annual thinning of pRNFL or mGCC, or annual loss of TMV among the three groups (Wald *p* > 0.05). However, the annual loss of pRNFL, mGCC, and TMV in the three groups was significantly greater than that in the HCs, respectively (Wald *p* < 0.05). Compared to HCs, the variation coefficient (β) and 95% CIs of the tocilizumab group were −1.53 (−2.79, −0.27; pRNFL), −1.29 (−2.71, 0.13; mGCC), and −0.103 (−0.201, −0.005; TMV). In the rituximab group, β and 95% CIs were −1.646 (−3.269, −0.024; pRNFL), −2.053 (−3.324, −0.782; mGCC), and −0.133 (−0.203, −0.063; TMV). In the azathioprine group, β and 95% CIs were −1.828 (−3.078, −0.577; pRNFL), −1.881 (−2.279, −1.484; mGCC), and −0.108 (−0.183, −0.033; TMV) (Figure 3).

Compared with baseline and HCs, NMOSD^ON+^ eyes revealed longitudinal optic disc cup area enlargement in the three groups of patients (annual increase 0.08 [−0.01, 0.16] mm^2^ in the tocilizumab group, 0.07 [0.01, 0.12] mm^2^ in the rituximab group, and 0.14 [0.02, 0.26] mm^2^ in the azathioprine group). In addition, NMOSD^ON+^ eyes also displayed an annual reduction of rim volume and increased cup volume in the three groups (*p* < 0.05). *Post-hoc* analysis did not reveal any differences in changes in disc cup area, cup volume, or rim volume, among the three groups. There were no significant changes in disc area, rim area, or nerve head volume in the three groups of patients with NMOSD, compared to HCs.

### Changes of OCT Measures in NMOSD^ON–^ Eyes During Follow-Ups

NMOSD^ON−^ eyes did not display mGCC and pRNFL thinning, or TMV loss compared with baseline in patients with tocilizumab and rituximab (*p* > 0.05). However, we observed significant loss of pRNFL (−5.34 [−2.87, −7.82] μm, *p* < 0.001), mGCC (−0.84 [−1.50, −0.18] μm, *p* = 0.017), and TMV (−0.09 [−0.14, −0.04] ${\text{mm}}_{,}^{3}$ *p* = 0.003) compared with baseline in patients treated with azathioprine (Table 4).

**Table 4:** Mean changes in the eyes without a history of ON in SD-OCT measures in patients with NMOSD and HCs.

	**Tocilizumab**		**Rituximab**		**Azathioprine**	
	**Eyes^**ON−**^**	***p***	**Eyes^**ON−**^**	***p***	**Eyes^**ON−**^**	***p***
**mGCC thickness (μm)**	−0.19 (−0.87, 0.48)	0.549	−0.07 (−1.25, −2.51)	0.846	−0.84 (−1.50, −0.18)	**0.017**
Superior	0.06 (−1.14, 0.88)	0.571	0.21 (−5.96, 3.09)	1	−1.10 –(2.2, 0.50)	0.107
Inferior	0.23 (−1.14,1.52)	1	−0.35 (−4.55, 1.94)	0.365	−0.83 (−3.21, 0.87)	0.169
FLV (%)	−0.06 (−0.25, 0.07)	0.498	0.06 (−0.39, 0.60)	0.898	−0.21 (−0.54, 0.86)	0.793
GLV (%)	0.63 (−0.30, 1.19)	0.075	1.16 (−1.18, 3.50)	0.295	0.58 (−0.43, 1.71)	0.135
**Global pRNFL (μm)**	−1.67 (−3.73, 0.40)	0.108	−2.67 (−6.45, 1.11)	0.147	−5.34 (−2.87, −7.82)	** <0.001**
**Macular**						
TMV (mm^3^)	−0.04 (−0.08, −0.01)	0.105	−0.07 (−0.14, 0.00)	0.059	−0.09 (−0.14, −0.04)	**0.003**
FT (μm)	−2.15 (−4.34, 0.03)	0.053	−1.4 (−3.74, 0.94)	0.209	−3.77 (−10.83, 3.292)	0.267
**Optic disc**						
Disc area (mm^2^)	−0.01 (−0.02, 0.00)	0.353	0.00 (−0.01, 0.02)	0.656	0.00 (−0.01, 0.02)	0.480
Cup area (mm^2^)	0.04 (0.01, 0.08)	**0.026**	0.03 (−0.03,0.08)	0.269	0.04 (−0.02, 0.10)	0.175
Cup volume (mm^3^)	−0.00 (−0.03, 0.02)	0.711	0.00 (−0.02, 0.03)	0.694	0.01 (−0.01, 0.03)	0.468
Rim area (mm^2^)	−0.03 (−0.06, 0.01)	0.098	−0.01 (−0.09, 0.03)	0.474	−0.18 (−0.50, 0.13)	0.234
Rim volume (mm^3^)	−0.01 (−0.03, 0.00)	0.096	−0.01 (−0.02, 0.00)	0.074	−0.01 (−0.02, 0.00)	0.087
Nerve head volume (mm^3^)	−0.01 (−0.04, 0.02)	0.242	−0.05 (−0.07, 0.03)	0.083	−0.01 (−0.05, 0.02)	0.519

*The differences were shown using B (lower CI, upper CI). FLV, focal loss volume; FT, fovea thickness; GLV, global loss volume; HCs, healthy controls; mGCC, macular ganglion cell complex; pRNFL, peripapillary retinal nerve fiber layer thickness; TMV, total macular volume. p-values that were <0.05 are in bold*.

When we compared the pRNFL, mGCC, and TMV annual changes in the three treatment groups with those in the HCs group, no significant differences were observed in the tocilizumab or the rituximab group. However, the NMOSD^ON−^ eyes treated with azathioprine displayed significantly reduced pRNFL (β −4.54 [−7.165, −1.923] μm, Wald *p* = 0.001), mGCC thickness (β −0.94 [−1.654, −0.230] μm, Wald *p* = 0.010), and TMV (β −0.08 [−0.144, −0.013] mm^3^, Wald *p* = 0.019) compared to HC eyes.

Patients treated with tocilizumab did not show any significant differences in annual changes of pRNFL, mGCC, and TMV in NMOSD^ON−^ eyes compared with patients treated with rituximab.

**Figure 4:**
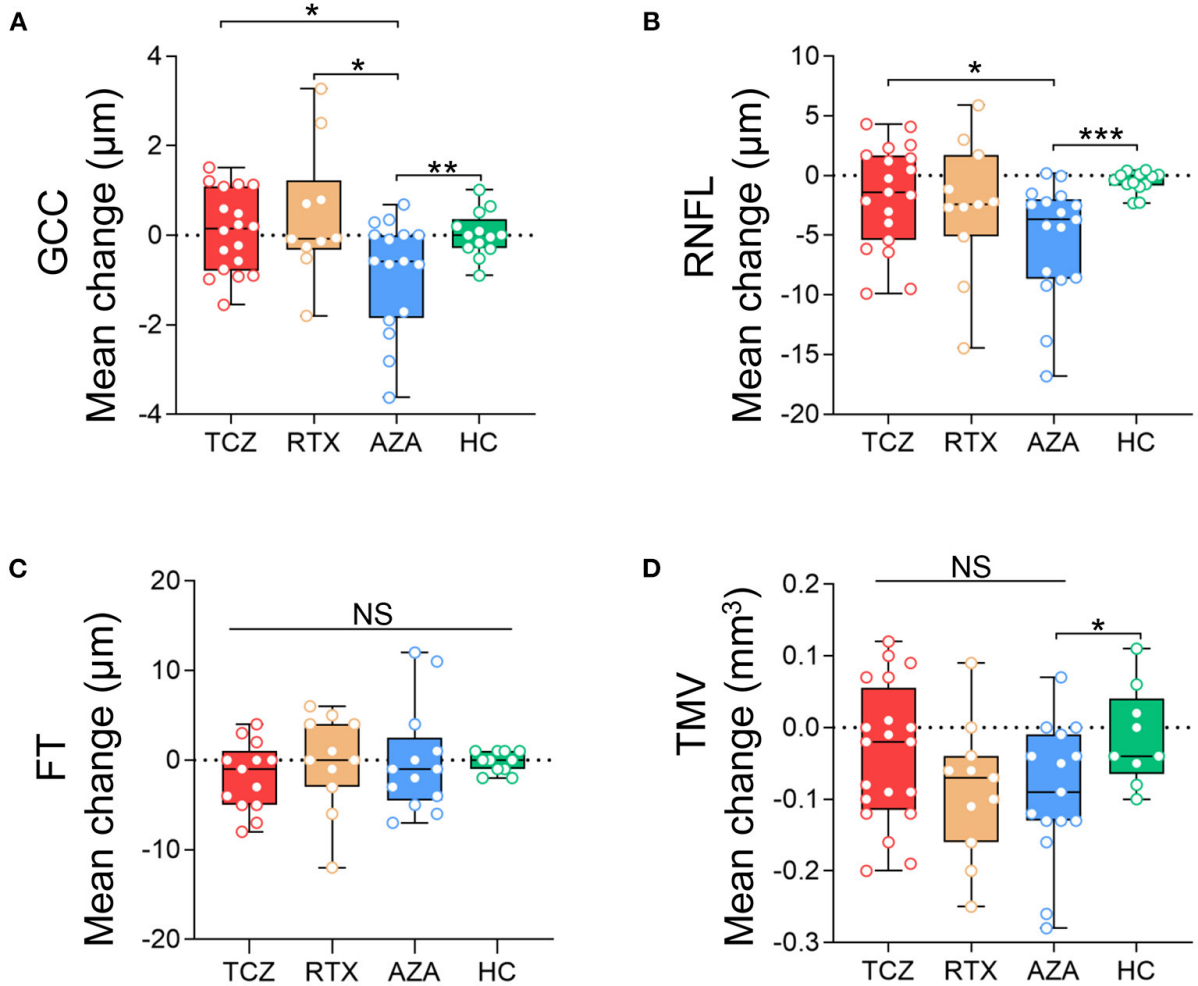
Comparison of the annual changes of SD-OCT measurements in NMOSDON- eyes in patients with NMOSD. Annual loss of the macular GCC **(A)**, RNFL **(B)**, FT **(C)**, and TMV **(D)** in eyes with a history of ON were compared between each two groups. GCC, ganglion cell complex; RNFL, retinal nerve fiber layer thickness; FT, fovea thickness; TMV, total macular volume; TCZ, tocilizumab; RTX, rituximab; AZA, azathioprine; HC, healthy control. *p < 0.05, **p < 0.01, ***p < 0.001. NS, not significant.

*Post-hoc* analyses also revealed that tocilizumab and rituximab significantly reduced mGCC annual thinning (β −1.00 [−1.779, −0.223] μm, Wald *p* = 0.012 and β −1.37 [−2.46, −0.27] μm, Wald *p* = 0.015, respectively) compared with azathioprine. Patients treated with tocilizumab (β −3.42 [−6.61, −0.23] μm, Wald *p* = 0.033) but not with rituximab treatment (β −2.41 [−6.50, 1.67] μm, Wald *p* = 0.089) had less pRNFL annual thinning compared with those treated with azathioprine. The annual change in TMV did not differ significantly between the three treatment groups (Figure 4).

We did not detect significant changes in FT or any disc measures in the three groups in NMOSD^ON−^ eyes compared with baseline or HCs.

## Discussion

Using SD-OCT with intra-retinal segmentation, we investigated longitudinal retinal layer changes in AQP4-IgG seropositive NMOSD^ON+^ eyes and NMOSD^ON−^ eyes under different treatments in comparison with matched HC eyes. While patients on immunosuppression had no relapses during the follow-up period, we observed longitudinal mGCC loss in NMOSD^ON+^ eyes of AQP4-IgG seropositive NMOSD patients, independent of suppressing activity. However, for NMOSD^ON−^ eyes, only those treated with azathioprine displayed significant mGCC loss compared to those treated with tocilizumab and rituximab treatment.

Consistent with previous OCT studies (3, 4, 6, 23), we observed retinal neuroaxonal degeneration in both NMOSD^ON+^ eyes and NMOSD^ON−^ eyes at baseline, displayed by reduced mGCC and thinning of pRNFL. mGCC reduction represents ganglion cell damage and pRNFL acts as a marker of retinal axonal loss. Reduced pRNFL and mGCC in NMOSD^ON+^ eyes reflected neuroaxonal degeneration. Neuroaxonal damage is more severe in NMOSD^ON+^ eyes than NMOSD^ON−^ eyes. This is paralleled with poorer visual acuity detected by LogMAR and low-contrast letter acuity. Poor visual function and prognosis may be associated with more severe neurodegeneration, including axonal accumulation of degenerative mitochondria with complement-dependent astrocyte pathology in ON lesions (24). Intriguingly, optic nerve involvement is not exclusive of nerves with prior ON. Astrocytic dysfunction in the retina leading to neuroaxonal damage and retrograde neuroaxonal degeneration may explain mGCC thinning in eyes without a history of ON (25, 26).

Evidence on subclinical retinal damage in NMOSD are very contradictory. Some studies did not detect differences in RNFL thickness between NMOSD^ON−^ eyes and healthy eyes (11, 27, 28). However, increasing evidence showed that subclinical retinal ganglion cell neuronal and axonal loss occurred in NMOSD^ON−^ eyes (7, 29). A comparative study also reported cases with no history of clinical ON but presence of RNFL and GCC loss in NMOSD, which indicates primary neuroaxonal pathology (24). Our data supported microstructural atrophy in NMOSD^ON−^ eyes (7). The retinal changes may be explained by AQP4-IgG-mediated pathology of astrocytes and Müller cells, regardless of ON history (24).

Previous studies revealed that neuroaxonal loss was very evident in the first 6 months in NMOSD^ON+^ eyes, and after the acute phase, there is no clear increase in neurodegeneration compared with NMOSD^ON−^ eyes (30). In our study, NMOSD^ON+^ eyes experienced progressive neuroaxonal degeneration over 6 months. This finding is consistent with findings of previous OCT studies also indicating that NMOSD patients display a progressive retinal thinning, regardless of disease activity (4, 5). Specifically, neuropathological findings show that AQP4 immunoreactivity on astrocytes is lost in anterograde/retrograde degeneration in the optic nerves. Similar to MS, the involvement of the anterior visual pathway in NMOSD is widespread and chronically progressive (31).

Although, tocilizumab and rituximab show high efficacy in reducing the risk of attacks in patients with NMOSD, neither of them can reverse axonal degeneration of mGCC and pRNFL after ON. In a case report, severe sensory impairment in a patient with NMOSD and intractable axonal neuropathy still gradually progressed during tocilizumab treatment (32). However, in an animal model of spinal cord injury pain, IL-6 signaling was predominantly elevated in reactive astrocytes and IL-6R blockade contributed to alleviating allodynia and decreasing glutamate transporter GLT-1 (33). Conflicting reports of the efficacy of tocilizumab in neurorepair warrants further confirmatory studies.

Different from previous studies, we investigated the influence of disease-modifying therapy on retinal changes. Although, we did not detect any differences in NMOSD^ON+^ eyes when comparing patients on biological agents vs. those on azathioprine and corticosteroids, we did observe axonal loss in eyes without prior ON in azathioprine-treated patients. We postulated that chronic neurodegeneration may persist in NMOSD^ON−^ eyes, as previous research indicates progressive neurodegeneration in AQP4-expressing brain structures. Cortical neuronal loss with unique AQP4 dynamics in astrocytes was also observed in pathological processes that could reflect progressive neurodegeneration in NMOSD (34). Besides NMOSD-related retinopathy, cytostatic or cytotoxic immunosuppressants, as well as corticosteroids, might induce neuronal damage (35). Another explanation is that azathioprine may be less effective compared with rituximab or tocilizumab in deterring neuroaxonal loss. AQP4-IgG itself has also been associated with progressive neurodegeneration in the visual system as well as in other CNS areas (24). Compared with the azathioprine-treated patients, rituximab is more effective in preventing relapses, and could improve the symptoms (36, 37). Rituximab also decreases AQP4-IgG titers more than azathioprine (38). The TANGO study showed that tocilizumab significantly reduces the risk of relapses and AQP4-IgG compared with azathioprine (17). Therefore, second-line treatments such as rituximab and tocilizumab may be more effective than azathioprine in suppressing this AQP4-IgG related neurodegeneration.

Patients in this study displayed no significant changes in LogMAR and low-contrast letter acuity in a median 1-year follow-up. Consistent with the visual acuity, TMV and FT remained lower compared to HCs in both NMOSD^ON+^ and NMOSD^ON−^ eyes compared to HC eyes and did not demonstrate longitudinal atrophy or improvement. This indicates that neurodegeneration is irreversible in NMOSD affected and unaffected eyes and early treatment of ON attacks is necessary, as subsequent visual acuity would not improve during the chronic remission period. Recent studies demonstrate that monoclonal antibodies (rituximab, eculizumab, inebilizumab, and satralizumab) were effective for the treatment of NMOSD (39); few studies focused on the changes of OCT measures and visual acuity. No difference was observed in changes in low-contrast letter acuity binocular score from baseline in an inebilizumab treatment group compared with placebo, though, inebilizumab significantly reduced the risk of an ON attack (40).

Earlier studies using optic disc photography revealed that pathological optic disc cupping was present in 16–37% of eyes affected by idiopathic ON (41). To the best of our knowledge, this is the first study to use OCT to determine if optic disc cupping occurs after ON in NMOSD. We found that after ON, optic disc cup area and volume were slightly enlarged in both NMOSD^ON+^ and NMOSD^ON−^ eyes. Optic disc cupping may progress slowly during follow-ups, explained by the loss of retinal nerve fibers. However, we observed no significant changes in optic nerve head topography measures, which we interpreted as a potential flooring effect resulting from previous ON episodes.

There were several limitations in this study. The follow-up period was relatively short, and each subgroup had a small sample size. Additionally, we were not able to analyze separate layers, such as the ganglion cell-inner plexiform layer using the RTVUE100-2 OCT instrument. The GCCs and pRNFLs with a history of severe ONs that were very thin and difficult to measure by other methods were excluded. Finally, this study lacks a rescanning protocol for OCT follow-ups.

## Conclusion

Our findings support the notion that disease-related damage occurs mainly during acute attacks. Chronic progressive retinopathy also occurs during remission. These findings suggest the importance of monitoring OCT measures to provide evidence for efficacious and safe disease-modifying therapy.

## Data Availability Statement

The original contributions presented in the study are included in the article/Supplementary Material, further inquiries can be directed to the corresponding author/s.

## Ethics Statement

The studies involving human participants were reviewed and approved by Institute Ethical Board of Tianjin Medical University General Hospital. The patients/participants provided their written informed consent to participate in this study.

## Author Contributions

CZ, PZ, and CD designed the study, collected, analyzed, and interpreted the data, and drafted and revised the manuscript. RZ provided technical support for the study. PZ and CD did statistical analysis. PZ, CD, DJ, FJ, MF, and CZ collected and analyzed the data and revised the manuscript critically for intellectual content. Authors involved in drafting the text and figures were CZ and PZ. All authors approved the final version of the manuscript.

## Conflict of Interest

The authors declare that the research was conducted in the absence of any commercial or financial relationships that could be construed as a potential conflict of interest.

## References

[1] Huda S Whittam D Bhojak M Chamberlain J Noonan C Jacob A. Neuromyelitis optica spectrum disorders. Clin Med. (2019) 19:169–76. 10.7861/clinmedicine.19-2-169 PMC6454358 30872305

[2] Henderson AP Altmann DR Trip AS Kallis C Jones SJ Schlottmann PG. A serial study of retinal changes following optic neuritis with sample size estimates for acute neuroprotection trials. Brain. (2010) 133:2592–602. 10.1093/brain/awq146 20566483

[3] Syc S Saidha S Newsome S Ratchford J Levy M Ford E. Optical coherence tomography segmentation reveals ganglion cell layer pathology after optic neuritis. Brain. (2012) 135:521–33. 10.1093/brain/awr264 22006982 PMC3281477

[4] Oertel FC Havla J Roca-Fernandez A Lizak N Zimmermann H Motamedi S. Retinal ganglion cell loss in neuromyelitis optica: a longitudinal study. J Neurol Neurosurg Psychiatry. (2018) 89:1259–65. 10.1136/jnnp-2018-318382 29921610

[5] Pisa M Ratti F Vabanesi M Radaelli M Guerrieri S Moiola L. Subclinical neurodegeneration in multiple sclerosis and neuromyelitis optica spectrum disorder revealed by optical coherence tomography. Mult Scler. (2020) 26:1197–206. 10.1177/1352458519861603 31392924

[6] Jeong IH Kim HJ Kim NH Jeong KS Park CY. Subclinical primary retinal pathology in neuromyelitis optica spectrum disorder. J Neurol. (2016) 263:1343–8. 10.1007/s00415-016-8138-8 27142716

[7] Oertel FC Kuchling J Zimmermann H Chien C Schmidt F Knier B. Microstructural visual system changes in AQP4-antibody-seropositive NMOSD. Neurol Neuroimmunol Neuroinflamm. (2017) 4:e334. 10.1212/NXI.0000000000000334 28255575 PMC5322864

[8] Gabilondo I Martinez-Lapiscina EH Martinez-Heras E Fraga-Pumar E Llufriu S Ortiz S. Trans-synaptic axonal degeneration in the visual pathway in multiple sclerosis. Ann Neurol. (2014) 75:98–107. 10.1002/ana.24030 24114885

[9] Tian DC Su L Fan M Yang J Zhang R Wen P. Bidirectional degeneration in the visual pathway in neuromyelitis optica spectrum disorder (NMOSD). Mult Scler. (2018) 24:1585–93. 10.1177/1352458517727604 28823217

[10] Naismith R Tutlam N Xu J Klawiter E Shepherd J Trinkaus K. Optical coherence tomography differs in neuromyelitis optica compared with multiple sclerosis. Neurology. (2009) 72:1077–82. 10.1212/01.wnl.0000345042.53843.d5 19307541 PMC2677471

[11] Ratchford J Quigg M Conger A Frohman T Frohman E Balcer L. Optical coherence tomography helps differentiate neuromyelitis optica and MS optic neuropathies. Neurology. (2009) 73:302–8. 10.1212/WNL.0b013e3181af78b8 19636050 PMC2843578

[12] Kim NH Kim HJ Park CY Jeong KS. Retinal degeneration after first-ever optic neuritis helps differentiate multiple sclerosis and neuromyelitis optica spectrum disorder. Front Neurol. (2019) 10:1076. 10.3389/fneur.2019.01076 31649616 PMC6795757

[13] Stangel M Kuhlmann T Matthews PM Kilpatrick TJ. Achievements and obstacles of remyelinating therapies in multiple sclerosis. Nat Rev Neurol. (2017) 13:742–54. 10.1038/nrneurol.2017.139 29146953

[14] Trebst C Jarius S Berthele A Paul F Schippling S Wildemann B. Update on the diagnosis and treatment of neuromyelitis optica: recommendations of the Neuromyelitis Optica Study Group (NEMOS). J Neurol. (2014) 261:1–16. 10.1007/s00415-013-7169-7 24272588 PMC3895189

[15] Damato V Evoli A Iorio R. Efficacy and safety of rituximab therapy in neuromyelitis optica spectrum disorders: a systematic review and meta-analysis. JAMA Neurol. (2016) 73:1342–8. 10.1001/jamaneurol.2016.1637 27668357

[16] Yamamura T Kleiter I Fujihara K Palace J Greenberg B Zakrzewska-Pniewska B. Trial of satralizumab in neuromyelitis optica spectrum disorder. N Engl J Med. (2019) 381:2114–24. 10.1056/NEJMoa1901747 31774956

[17] Zhang C Zhang M Qiu W Ma H Zhang X Zhu Z. Safety and efficacy of tocilizumab versus azathioprine in highly relapsing neuromyelitis optica spectrum disorder (TANGO): an open-label, multicentre, randomised, phase 2 trial. Lancet Neurol. (2020) 19:391–401. 10.1016/S1474-4422(20)30070-3 32333897 PMC7935423

[18] Wingerchuk D Banwell B Bennett J Cabre P Carroll W Chitnis T. International consensus diagnostic criteria for neuromyelitis optica spectrum disorders. Neurology. (2015) 85:177–89. 10.1212/WNL.0000000000001729 26092914 PMC4515040

[19] Raftopoulos R Hickman SJ Toosy A Sharrack B Mallik S Paling D. Phenytoin for neuroprotection in patients with acute optic neuritis: a randomised, placebo-controlled, phase 2 trial. Lancet Neurol. (2016) 15:259–69. 10.1016/S1474-4422(16)00004-1 26822749

[20] Cruz-Herranz A Balk L Oberwahrenbrock T Saidha S Martinez-Lapiscina E Lagreze W. The APOSTEL recommendations for reporting quantitative optical coherence tomography studies. Neurology. (2016) 86:2303–9. 10.1212/WNL.0000000000002774 27225223 PMC4909557

[21] Tewarie P Balk L Costello F Green A Martin R Schippling S. The OSCAR-IB consensus criteria for retinal OCT quality assessment. PLoS ONE. (2012) 7:e34823. 10.1371/journal.pone.0034823 22536333 PMC3334941

[22] Yang C Yang L Li T Zhang D Jin W Li M. Responsiveness to reduced dosage of rituximab in Chinese patients with neuromyelitis optica. Neurology. (2013) 81:710–3. 10.1212/WNL.0b013e3182a1aac7 23884041 PMC3776460

[23] Akaishi T Kaneko K Himori N Takeshita T Takahashi T Nakazawa T. Subclinical retinal atrophy in the unaffected fellow eyes of multiple sclerosis and neuromyelitis optica. J Neuroimmunol. (2017) 313:10–5. 10.1016/j.jneuroim.2017.10.001 29153600

[24] Hokari M Yokoseki A Arakawa M Saji E Yanagawa K Yanagimura F. Clinicopathological features in anterior visual pathway in neuromyelitis optica. Ann Neurol. (2016) 79:605–24. 10.1002/ana.24608 26836302

[25] Felix CM Levin MH Verkman AS. Complement-independent retinal pathology produced by intravitreal injection of neuromyelitis optica immunoglobulin G. J Neuroinflammation. (2016) 13:275. 10.1186/s12974-016-0746-9 27765056 PMC5072328

[26] Kurosawa K Misu T Takai Y Sato DK Takahashi T Abe Y. Severely exacerbated neuromyelitis optica rat model with extensive astrocytopathy by high affinity anti-aquaporin-4 monoclonal antibody. Acta Neuropathol Commun. (2015) 3:82. 10.1186/s40478-015-0259-2 26637322 PMC4670539

[27] de Seze J Blanc F Jeanjean L Zephir H Labauge P Bouyon M. Optical coherence tomography in neuromyelitis optica. Arch Neurol. (2008) 65:920–3. 10.1001/archneur.65.7.920 18625858

[28] Schneider E Zimmermann H Oberwahrenbrock T Kaufhold F Kadas EM Petzold A. Optical coherence tomography reveals distinct patterns of retinal damage in neuromyelitis optica and multiple sclerosis. PLoS ONE. (2013) 8:e66151. 10.1371/journal.pone.0066151 23805202 PMC3689687

[29] Filippatou AG Vasileiou ES He Y Fitzgerald KC Kalaitzidis G Lambe J. Evidence of subclinical quantitative retinal layer abnormalities in AQP4-IgG seropositive NMOSD. Mult Scler. (2020). 10.1177/1352458520977771. [Epub ahead of print]. 33307967 PMC8200372

[30] Manogaran P Traboulsee A Lange A. Longitudinal study of retinal nerve fiber layer thickness and macular volume in patients with neuromyelitis optica spectrum disorder. J Neuroophthalmol. (2016) 36:363–8. 10.1097/WNO.0000000000000404 27416520

[31] Talman L Bisker E Sackel D Long D Galetta K Ratchford J. Longitudinal study of vision and retinal nerve fiber layer thickness in multiple sclerosis. Ann Neurol. (2010) 67:749–60. 10.1002/ana.22005 20517936 PMC2901775

[32] Mizuno Y Shinoda K Watanabe M Ogata H Isobe N Matsushita T. Intractable axonal neuropathy with multifocal peripheral nerve swelling in neuromyelitis optica spectrum disorders: a case report. Mult Scler Relat Disord. (2019) 35:16–8. 10.1016/j.msard.2019.06.033 31279231

[33] Guptarak J Wanchoo S Durham-Lee J Wu Y Zivadinovic D Paulucci-Holthauzen A. Inhibition of IL-6 signaling: a novel therapeutic approach to treating spinal cord injury pain. Pain. (2013) 154:1115–28. 10.1016/j.pain.2013.03.026 23639820

[34] Saji E Arakawa M Yanagawa K Toyoshima Y Yokoseki A Okamoto K. Cognitive impairment and cortical degeneration in neuromyelitis optica. Ann Neurol. (2013) 73:65–76. 10.1002/ana.23721 23378324

[35] Diem R Hobom M Maier K Weissert R Storch MK Meyer R. Methylprednisolone increases neuronal apoptosis during autoimmune CNS inflammation by inhibition of an endogenous neuroprotective pathway. J Neurosci. (2003) 23:6993–7000. 10.1523/JNEUROSCI.23-18-06993.2003 12904460 PMC6740669

[36] Nikoo Z Badihian S Shaygannejad V Asgari N Ashtari F. Comparison of the efficacy of azathioprine and rituximab in neuromyelitis optica spectrum disorder: a randomized clinical trial. J Neurol. (2017) 264:2003–9. 10.1007/s00415-017-8590-0 28831548

[37] Zhang M Zhang C Bai P Xue H Wang G. Effectiveness of low dose of rituximab compared with azathioprine in Chinese patients with neuromyelitis optica: an over 2-year follow-up study. Acta Neurol Belg. (2017) 117:695–702. 10.1007/s13760-017-0795-6 28608315

[38] Yang Y Wang CJ Wang BJ Zeng ZL Guo SG. Comparison of efficacy and tolerability of azathioprine, mycophenolate mofetil, and lower dosages of rituximab among patients with neuromyelitis optica spectrum disorder. J Neurol Sci. (2018) 385:192–7. 10.1016/j.jns.2017.12.034 29406904

[39] Xue T Yang Y Lu Q Gao B Chen Z Wang Z. Efficacy and safety of monoclonal antibody therapy in neuromyelitis optica spectrum disorders: evidence from randomized controlled trials. Mult Scler Relat Disord. (2020) 43:102166. 10.1016/j.msard.2020.102166 32442886

[40] Cree BAC Bennett JL Kim HJ Weinshenker BG Pittock SJ Wingerchuk DM. Inebilizumab for the treatment of neuromyelitis optica spectrum disorder (N-MOmentum): a double-blind, randomised placebo-controlled phase 2/3 trial. Lancet. (2019) 394:1352–63. 10.1016/S0140-6736(19)31817-3 31495497

[41] Trip S Schlottmann P Jones S Garway-Heath D Thompson A Plant G. Quantification of optic nerve head topography in optic neuritis: a pilot study. Br J Ophthalmol. (2006) 90:1128–31. 10.1136/bjo.2006.092031 16774960 PMC1857379

