# Supplementary material for: Optical Coherence Tomography Reveals Longitudinal Changes in Retinal Damage Under Different Treatments for Neuromyelitis Optica Spectrum Disorder
Source: Front Neurol. 2021 Jul 19;12:669567. doi: 10.3389/fneur.2021.669567 (PMC8326361; doi:10.3389/fneur.2021.669567)
Supplement: Supplementary file 1 [file Table_1.DOCX]

**Supplementary Figures.**

B

A


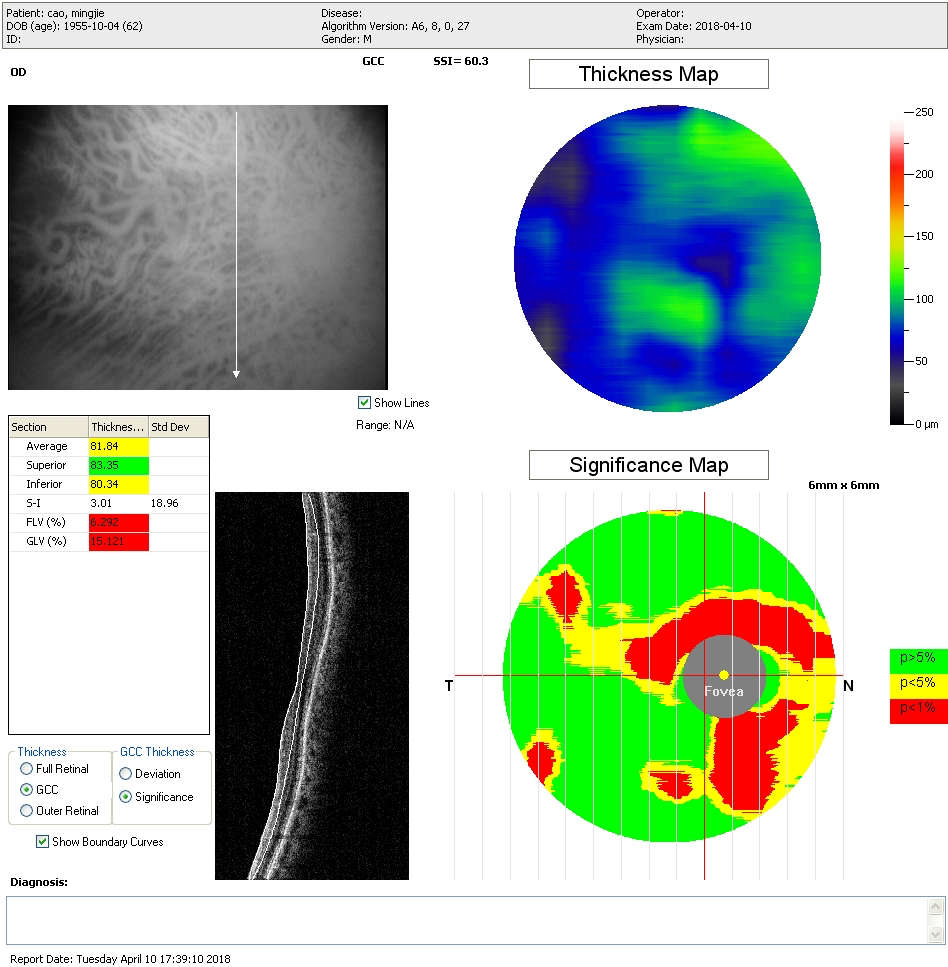
**
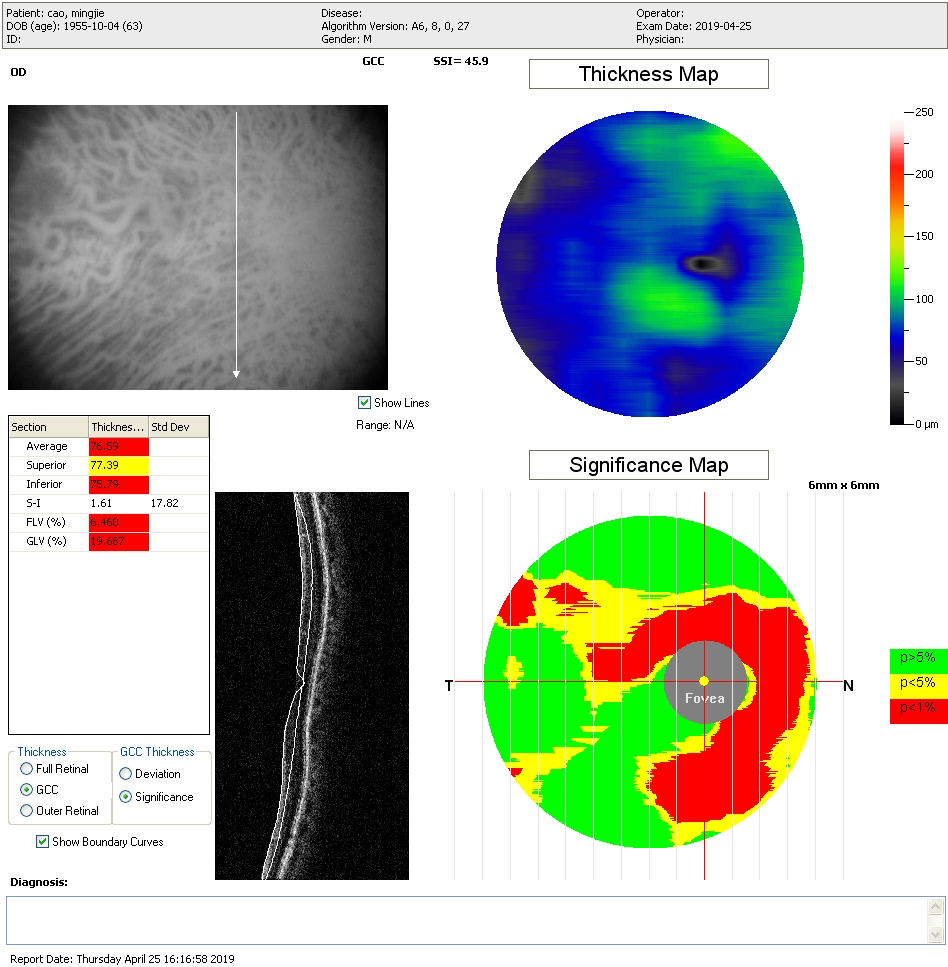
**

**
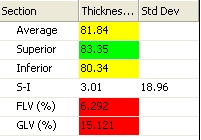

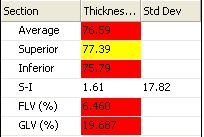
**

**Figure 1. The scanning of the macular ganglion cell complex in one eye of a patient with NMOSD receiving tocilizumab treatment.** The thickness maps of macular ganglion cell complex (mGCC) at baseline (A) and 1 year later (B) were shown. The quadrant thickness was shown under the image, which were calculated automatically.

B

A


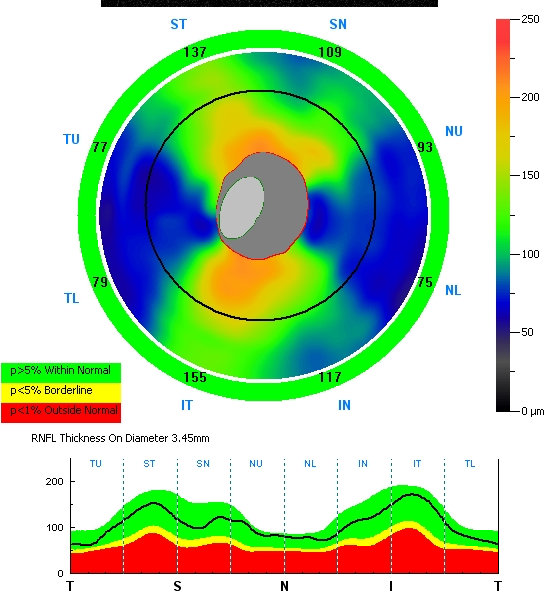

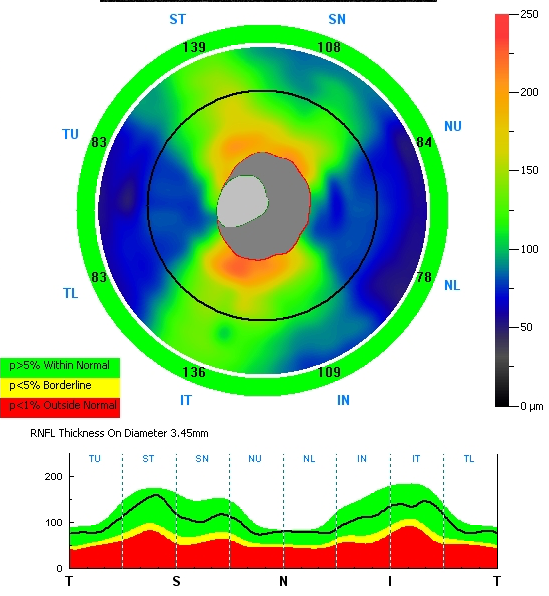


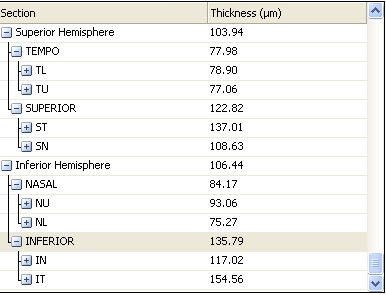

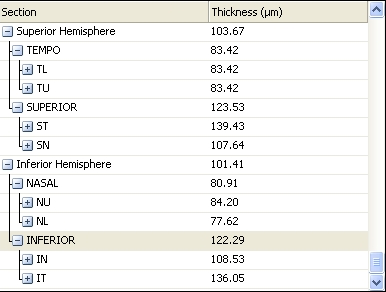


**Figure 2. The scanning of retinal nerve fiber layer thickness in one eye of a patient with NMOSD receiving tocilizumab treatment.** The thickness maps of retinal nerve fiber at baseline (A) and 1 year later (B) were shown. The quadrant thickness was under the image, which were calculated automatically.

B

A


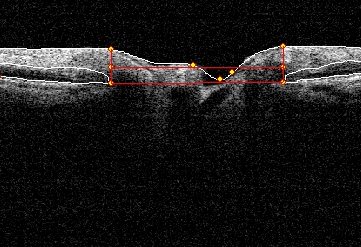

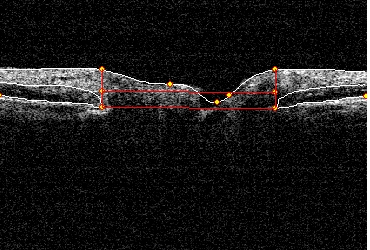


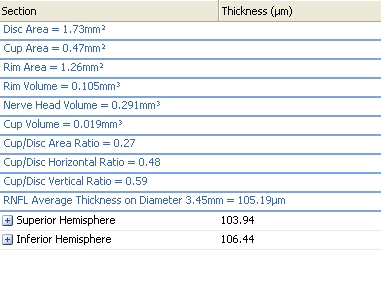

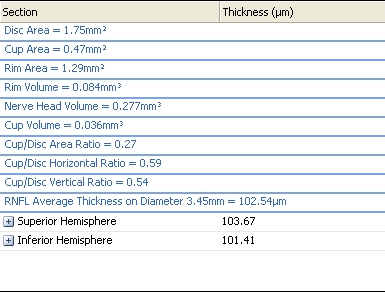


**Figure 3. The scanning of the optic disc in one eye of a patient with NMOSD receiving tocilizumab treatment.** The optic disc was shown at baseline (A) and 1 year later (B), respectively. The index was under the image, which were calculated automatically.
