# Supplementary material for: Optical Coherence Tomography Reveals Longitudinal Changes in Retinal Damage Under Different Treatments for Neuromyelitis Optica Spectrum Disorder
Source: Front Neurol. 2021 Jul 19;12:669567. doi: 10.3389/fneur.2021.669567 (PMC8326361; doi:10.3389/fneur.2021.669567)
Supplement: Supplementary file 2 [file Table_2.DOCX]

**Supplementary** **Table 1. Baseline OCT measures in patients with NMOSD and HCs.**

|  | **Tocilizumab** | | **Rituximab** | | | **Azathioprine** | | | **HCs** | |
| --- | --- | --- | --- | --- | --- | --- | --- | --- | --- | --- |
|  | **Eyes^ON+^** | **Eyes^ON-^** | | **Eyes^ON+^** | **Eyes^ON-^** | | **Eyes^ON+^** | **Eyes^ON-^** | | **Mean (SD)** |
| **mGCC thickness (μm)** | 71.68 (7.50) | 93.20 (3.38) | | 69.47 (6.52) | 90.65 (3.11) | | 64.59 (7.65) | 91.27 (3.64) | | 97.53 (2.32) |
| Superior | 78.28 (15.79) | 94.17 (4.81) | | 69.71 (7.83) | 86.21 (6.95) | | 63.31 (6.82) | 90.76 (4.86) | | 94.35 (4.21) |
| Inferior | 73.20 (11.08) | 93.12 (3.87) | | 70.48 (7.07) | 86.99 (7.39) | | 60.87 (6.52) | 90.83 (5.16) | | 94.37 (3.67) |
| FLV (%) | 6.99 (5.68) | 0.58 (0.45) | | 5.86 (1.97) | 1.73 (2.75) | | 7.66 (4.86) | 1.49 (1.42) | | 0.45 (0.49) |
| GLV (%) | 21.28 (10.37) | 4.80 (2.88) | | 26.10 (7.51) | 10.30 (6.75) | | 31.17 (8.80) | 6.57 (3.20) | | 4.59 (1.85) |
| **Global pRNFL (μm)** | 69.62 (11.64) | 112.10 (8.37) | | 77.11 (13.03) | 106.90 (4.70) | | 69.47 (14.12) | 111.10 (5.79) | | 112.40 (8.10) |
| TL pRNFL | 58.90 (14.07) | 88.60 (6.00) | | 58.01 (9.91) | 81.67 (10.45) | | 46.92 (9.80) | 83.95 (13.36) | | 87.41 (9.90) |
| TU pRNFL | 63.75 (15.12) | 89.66 (9.21) | | 57.45(12.69) | 80.44 (8.12) | | 51.13 (11.93) | 89.43 (13.54) | | 84.77 (7.76) |
| ST pRNFL | 101.4 (23.99) | 145.4 (15.34) | | 102.1 (19.10) | 144.9 (12.17) | | 87.34 (17.81) | 145.9 (21.14) | | 143.1 (19.14) |
| SN pRNFL | 92.95 (22.17) | 124.1 (20.45) | | 88.97 (14.78) | 129.2 (11.76) | | 86.61 (17.45) | 127.3 (17.93) | | 119.7 (11.78) |
| NU pRNFL | 57.84 (14.47) | 87.55 (1.731) | | 64.98 (13.52) | 88.30 (4.56) | | 50.41 (19.25) | 86.39 (10.35) | | 85.17 (11.31) |
| NL pRNFL | 51.89 (9.43) | 74.74 (14.74) | | 52.92 (10.41) | 70.36 (11.57) | | 48.36 (13.36) | 73.66 (8.404) | | 76.66 (5.328) |
| IN pRNFL | 79.70 (11.33) | 122.3 (12.25) | | 83.32 (12.24) | 110.5 (14.01) | | 70.98 (10.31) | 118.6 (14.91) | | 115.6 (11.82) |
| IT pRNFL | 100.2 (23.12) | 162.1 (14.97) | | 102.1 (19.92) | 148.3 (7.42) | | 84.61 (19.33) | 150.9 (15.57) | | 151.9 (13.77) |
| **Fovea** |  |  | |  |  | |  |  | |  |
| TMV (mm^3^) | 6.41 (0.36) | 6.89 (0.22) | | 6.37 (0.20) | 6.87 (0.16) | | 6.13 (0.26) | 6.82 (0.27) | | 7.23 (0.37) |
| FT (μm) | 229.4 (12.73) | 241.3 (14.16) | | 225.7 (15.20) | 239.3 (13.15) | | 227.3 (10.43) | 225.4 (9.71) | | 252.73 (18.58) |
| FV (mm^3^) | 0.17 (0.01) | 0.18 (0.01) | | 0.18 (0.01) | 0.18 (0.01) | | 0.18 (0.01) | 0.18 (0.01) | | 0.19 (0.20) |
| **Optic disc** |  |  | |  |  | |  |  | |  |
| Disc area (mm^2^) | 2.35 (0.52) | 2.17 (0.37) | | 2.48 (0.48) | 2.10 (0.31) | | 2.28 (0.52) | 2.14 (0.32) | | 2.25 (0.38) |
| Cup area (mm^2^) | 1.18 (0.75) | 0.64 (0.31) | | 1.32 (0.64) | 0.56 (0.25) | | 1.08 (0.61) | 0.65 (0.36) | | 0.61 (0.37) |
| Cup volume (mm^3^) | 0.25 (0.17) | 0.10 (0.07) | | 0.25 (0.21) | 0.08 (0.05) | | 0.21 (0.14) | 0.11 (0.09) | | 0.10 (0.09) |
| Rim area (mm^2^) | 1.18 (0.47) | 1.53 (0.23) | | 1.16 (0.53) | 1.55 (0.27) | | 1.20 (0.34) | 1.49 (0.35) | | 1.70 (0.34) |
| Rim volume (mm^3^) | 0.09 (0.09) | 0.10 (0.06) | | 0.08 (0.07) | 0.19 (0.08) | | 0.08 (0.05) | 0.17 (0.07) | | 0.23 (0.12) |
| Nerve head volume (mm^3^) | 0.17 (0.11) | 0.35 (0.09) | | 0.20 (0.15) | 0.38 (0.18) | | 0.20 (0.10) | 0.32 (0.13) | | 0.44 (0.23) |

Data were shown using mean (SD). HCs, healthy controls.
